# Bone cement leaking into iliac vein during artificial femoral head replacement: A case report

**DOI:** 10.1097/MD.0000000000017547

**Published:** 2019-10-11

**Authors:** Zhencun Cai, Chengzhe Piao, Ming Sun, Hongyu Zhou, Zhenhuai Gao, Liangbi Xiang

**Affiliations:** a Department of Orthopaedics Surgery, Central Hospital of Shenyang Medical College; b Department of Orthopaedics Surgery, General Hospital of Northern Theater Command, Shenyang, Liaoning Province, China.

**Keywords:** femoral neck fracture, hip hemiarthroplasty, iliac vessels, leaked bone cement

## Abstract

**Rationale:**

Leakage of bone cement from femoral medullary cavity is a rare complication after hip arthroplasty, and there is no report on the leaked bone cement entering into iliac vessels.

**Patient concerns:**

An 89-year-old woman presented with a fracture in the right femoral neck. She had well-fixed right femoral head replacement after careful preoperative examinations, and no adverse reactions appeared. She was able to get off bed to walk at the 2nd day after surgery.

**Diagnoses:**

Postoperative radiograph showed leakage of bone cement into the joint through femoral medullary cavity entering into iliac vessels, but the patient complained no discomforts. She received a treatment with low-molecular weight heparin and rivaroxaban.

**Outcomes:**

The patient was able to walk with normal gait, without swelling in both lower extremities and discomfort in the hip. There was no other complication concerning intravascular foreign bodies.

**Lessons:**

This case calls into the phenomenon of leakage of injected bone cement in femoral head replacement regardless of complete and nonfractured femur, which may be into the lower limb and pelvic veins, given that, dangerous consequences will not occur.

## 1 Introduction

As the proportion of the elderly increases, femoral neck fractures are becoming more and more common in life.^[1]^ Artificial femoral head replacement is an effective treatment for femoral neck fractures in the elderly, which has been unanimously recognized by scholars.^[2,3]^ There may be some complications during artificial femoral head replacement, but it is very rare to have bone cement leaking out of femoral medullary cavity. No case of bone cement leaking into iliac vein has been reported. We encountered a case of bone cement leaking into iliac vein during artificial femoral head replacement, which is reported in the following section.

## 2 Case report

**Figure 1:**
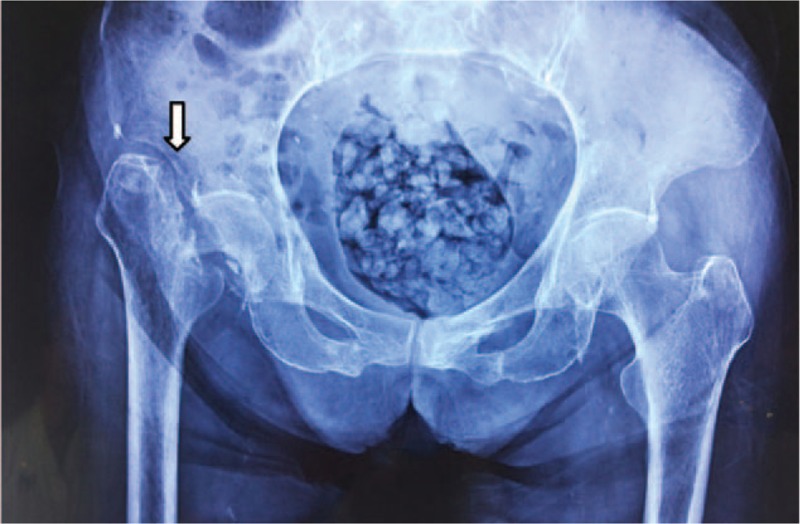
Preoperative X-ray film of bilateral hip joints. Right femoral neck presents with fracture, without abnormal changes in the regions surrounding femur or vascular running.

The patient, an 89-year-old woman, came to our hospital because of pain and 2-month limited mobility in her right hip joint after a fall. Before the injury, the patient was able to walk normally and had no history of other diseases. Two months ago, the patient accidentally fell while walking, and her right hip joint landed 1st during the fall. After the injury, she had pain, limited mobility, and inability to stand and bear weight in her right hip joint. Afterwards, she was laid up at home, without receiving any diagnosis and treatment. Because the condition was not getting better, the patient came to our hospital for examination. Physical examination on admission revealed that: both lower extremities were not swollen, the skin color was normal, and right lower extremity turned outwards for 45° and was 1 cm shorter than left lower extremity; the muscle strength of both lower extremities was at level 5, and the hip, knee, and ankle joints of the left lower extremity were all in normal mobility; there is limited mobility and palpable sensation of fracture friction in right hip joint, with flexion 60°, retroextension 0°, abduction 10°, adduction 5°, outward turning 10°, and inward turning 0°, and the knee and ankle joints are in normal mobility; the dorsalis pedis arteries of both lower extremities pulsed well, and the skin pain and temperature sensation were normal; X-ray examination of bilateral hip joints showed that the patient's bone density had decreased, the bone cortex continuity of the right femoral neck was interrupted, partial bone resorption occurred at the femoral neck and femoral head, the right Shenton line was discontinuous, and right femoral greater trochanter had moved up (Fig. 1). Admission diagnosis was as follows: right femoral neck fracture and osteoporosis.

**Figure 2:**
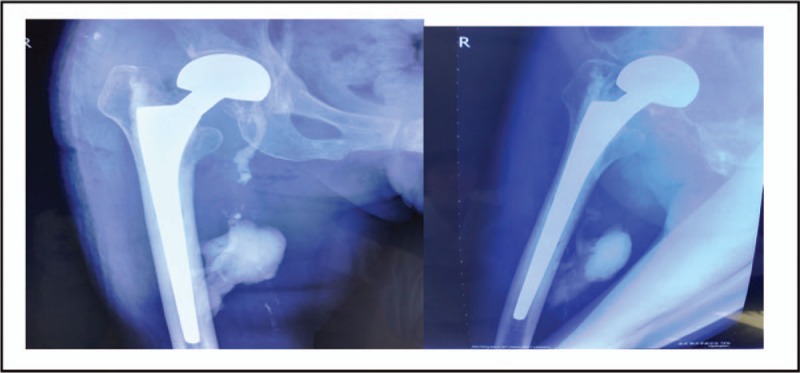
Anterioposterior and lateral X-ray films of right hip joint at postoperative 1 day. Femoral head prosthesis is in good position and shows no fracture. A high-density mass bone cement shadow is visible at the medial side of femur, and a strip-like bone cement shadow appears at the region of femoral vein running.

**Figure 3:**
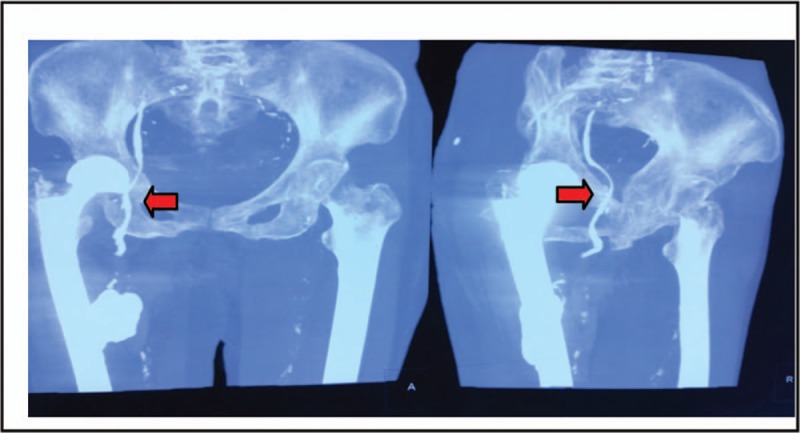
Postoperative computed tomography images. Bone cement mass locates at the posteromedial side of proximal femur, and continuous strip-like bone cements are found in the femoral and iliac veins.

**Figure 4:**
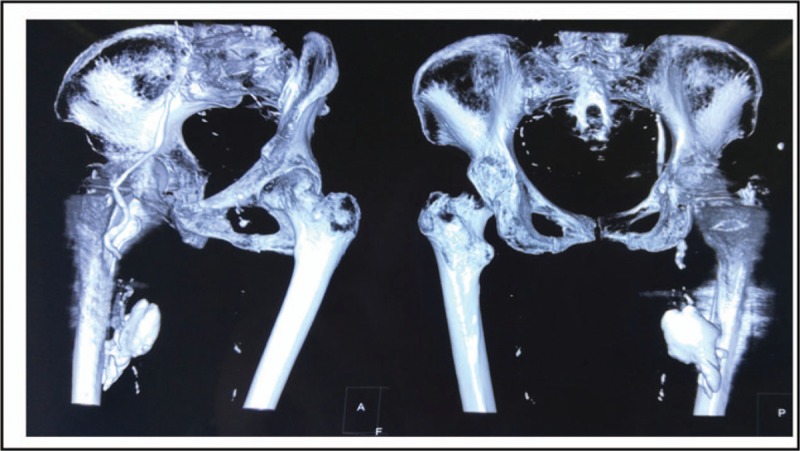
Postoperative 3-dimensional computed tomography reconstruction images. Femoral cortex is consecutive. The leaked bone cement extends to the pelvic cavity.

**Figure 5:**
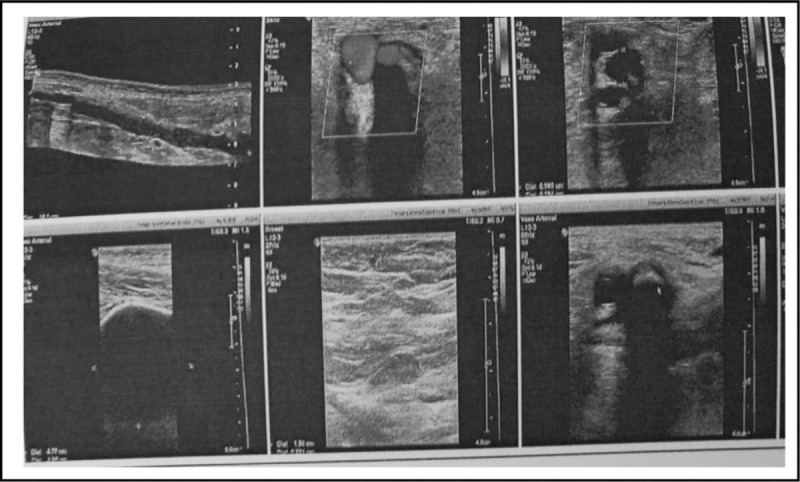
Postoperative color-Doppler ultrasound images of vessels. Bone cement presents along the intima of femoral and iliac vein wall, which causes luminal stenosis, but blood flow is fluent.

After admission, we conducted comprehensive physical examination, electrocardiogram, cardiac ultrasound, arteriovenous ultrasonography of both lower extremities on the patient before the operation, and no abnormalities were found. On the 3rd day after admission, we treated the patient with artificial femoral head replacement through the posterolateral approach of the hip joint. Considering the patient's osteoporosis, we injected 10 g bone cement into the femoral medullary cavity to fix the femoral stem prosthesis. The operation course was smooth, and the patient's vital signs remained stable: the ECG monitoring indexes were normal, the blood oxygen saturation was 97% to 99%, the heart rate was at 80 to 90 beats/min, the breathing rate was at 16 to 20 beats/min, and the patient had no obvious discomfort in the heart and respiratory system. On the 1st day after operation, the patient's mental state was good, the surgical incision was not red and swollen, both lower extremities were not swollen, and the skin color was normal. With the aid of walking aids, the patient could walk on her own. We performed routine double-hip X-ray examination on the patient and found that the femoral head prosthesis was in good position and the femur was in good shape. However, the image of a massive, dense foreign body, 4 × 4 × 5 cm^3^ in size, was seen in muscle gap outside the right femoral medullary cavity. Besides, in the inguinal region, there was a high-density strip-like image along the blood vessel, about 0.8 cm in diameter, extending to the pelvic cavity. Density of the foreign body image was the same as that of the bone cement (Fig. 2). We further performed computed tomography and vascular ultrasonography on the patient, through which we confirmed that the femoral cortex was good, without fracture, there was a bone cement mass in the muscle gap outside the femoral medullary cavity, in the right femoral vein and external iliac vein, along the intima of vein wall, there was a sleeve-like bone cement, making the venous lumen narrow (Figs. 3–5). We 1st adopted low-molecular weight heparin (LMWH) anticoagulant therapy (41 million IU, subcutaneous injection, once a day) on the patient; and then, 2 weeks later, we used rivaroxaban (10 mg, orally, once a day) for the further treatment. The patient was followed up for 1 year. During the follow-up, the vascular ultrasonography showed that the status of the bone cement in the iliac vein had no change, and the blood flow was fluent. The patient was able to walk with normal gait, without swelling in both lower extremities, and discomfort in the hip.

## 3 Discussion

Femoral neck fractures often occur in the elderly. Artificial femoral head replacement has the characteristics of small trauma, short operation time, and less intraoperative blood loss, so that it has been widely used on the patients with advanced age, low mobility before injury, and difficulty in tolerating large trauma.^[4,5]^ Calcium loss increases with age, and along with the increase of age, the calcium absorption capacity gradually decreases, so osteoporosis is common in the elderly.^[6]^ During artificial femoral head replacement, it is difficult to directly fix the osteoporotic femur with the biofemoral stem prosthesis, so for patients with osteoporosis, we often use cement-type femoral stem prosthesis. After the bone cement injected into the femoral medullary cavity is hardened, the femoral stem can be well bonded to the femur, and in this way, the femoral stem can be fixed. Gjertsen et al found that the rate of reoperation after uncemented hemiarthroplasty was significantly higher than that after cemented hemiarthroplasty.^[7]^ A 12- to 19-year follow-up study of elderly patients over 75 years found that cemented hemiarthroplasty had a much lower rate of reoperation than uncemented hemiarthroplasty.^[8]^ Morris study supports the use of modern cemented prosthesis as opposed to modern uncemented hemiarthroplasty. Postoperative complication rate after uncemented prosthesis is unacceptably high, especially periprosthetic fracture rate.^[9]^ It has been reported in the literature that the application of bone cement in femoral head replacement may cause tiny particles of bone cement to enter the blood circulation, leading to allergy and other adverse reactions,^[10]^ but there have been no reports on a large number of solid cement penetrating the complete femoral cortex and leaking to the muscle gap to form solid, let alone the reports on bone cement entering the blood vessels to form vascular sleeves.

Previous literatures reported that the leakage of bone cement out of bone is closely related to the distribution of blood vessels around bone,^[11,12]^ but it is more common in percutaneous vertebroplasty. After the bone cement is injected into the vertebral body, the vertebral body can maintain stable state to reduce the second fracture, and meanwhile, thermal effect can be produced to effectively kill the cells of peripheral nerve of vertebral body, reducing the pain.^[13,14]^ Due to the rich venous distribution in the spine, after entering the vertebral body, the bone cement will flow into the extraspinal venous system through the intraspinal venous system, causing the leakage of bone cement. In addition, vertebral bone cortex of the patient undergoing vertebroplasty suffers from a certain degree of damage, so the bone cement injected into the vertebral body can leak out of the vertebral body through the ruptured cortex.^[15]^ However, there is no abundant venous system around the femoral shaft, and the patient undergoing artificial femoral head replacement does not suffer from femoral cortical destruction and fracture, so it is difficult for bone cement to seep out of the femur, not to mention having access to the femoral or iliac veins.

So, in this case, how does the bone cement leak to the muscle gap and enter the blood vessels? We speculated that there are very few femur nourishing blood vessels, but after the bone cement was injected into the medullary cavity, the semi-liquid bone cement that had not hardened still entered the femur nourishing blood vessels due to excessive pressure in the femoral medullary cavity, and then flowed to the outside of femur along the blood vessels, resulting in rupture of blood vessels, and then flowed out of the ruptured blood vessels and formed a mass in the muscle gap. Along with the further leakage of bone cement, the pressure of massive bone cement continued to increase, and semi-solid bone cement further entered the branch vessels of intermuscular femoral vein and then entered the femoral vein and external iliac vein along the direction of blood flow. During the flowing, bone cement gradually adhered to the inner wall of blood vessels and extended to a distance. When the cement solidification time was reached, the cement entering the muscle gap formed solid mass, and the cement entering the blood vessels solidified to form solid vascular sleeves.

Donaldson et al and Holt et al found that the use of bone cement in artificial joint replacement can increase mortality, based on the fact that the chemical reaction of bone cement can increase the risk of blood pressure instability after the injection of bone cement into the medullary cavity, which further causes heart events and leads to an increase in mortality.^[16,17]^ In the past, it is believed that if bone cement enters the blood vessels, solid masses may form in the blood vessels after solidification, leading to bone cement embolism, blood flow interruption and tissue infarction.^[18,19]^ However, in this case, we found that the bone cement entering the blood vessels did not immediately form a mass, but was distributed along the inner wall of blood vessel in the direction of blood flow, forming a thin lamellar structure attached to the inner wall of the blood vessel. The reason for this may be that the liquid blood and the semi-solid bone cement could not dissolve in each other, and the bone cement that entered the blood vessel also had a certain degree of fluidity, so that under the impact of blood flow, the bone cement that did not dissolve in the blood flowed and solidified along the inner wall of blood vessel. Therefore, the bone cement entering the blood vessels did not immediately cause vascular blockage and blood flow interruption after solidification, and instead, it formed a vascular sleeve that narrows the blood vessel. In this case, within 1 year after operation, there was no obvious vascular embolism in the patient, the patient's limbs were not swollen and had good mobility, and the bone cement in the blood vessels did not fall off to cause embolization of other organs. All these indicate that the solidified bone cement entering the blood vessels may not cause significant blood coagulation reaction, and the bone cement in the blood vessels does not fall off easily after solidification.

The purpose of this case report is to remind everyone that in the process of using bone cement for artificial femoral head replacement, bone cement can leak out of the femur and may enter the veins of lower extremities. We need to properly control the bone cement injection time and pressure during the operation. Besides, this case also shows that if bone cement enters the vascular system in semi-solid state, it will not immediately form a mass to result in embolism of blood vessels, but form a thin vascular sleeve along the inner wall of blood vessel.

## Author contributions

**Conceptualization:** Liangbi Xiang, Zhencun Cai.

**Data curation:** Zhencun Cai, Ming Sun, Hongyu Zhou, Zhenhuai Gao.

**Methodology:** Chengzhe Piao.

**Supervision:** Zhencun Cai, Chengzhe Piao.

**Validation:** Zhencun Cai.

**Writing – original draft:** Zhencun Cai.

**Writing – review & editing:** Zhencun Cai.
